# The influence of oestrous substances on cyclicity and oestrous behaviour in dairy heifers

**DOI:** 10.1186/1751-0147-54-26

**Published:** 2012-04-17

**Authors:** Kristina Nordéus, Renée Båge, Hans Gustafsson, Patrice Humblot, Lennart Söderquist

**Affiliations:** 1 Department of Clinical Sciences, Division of Reproduction, Swedish University of Agricultural Sciences, P.O. Box 7054, SE-750 07 Uppsala, Sweden; 2 Swedish Dairy Association, P.O. Box 210, SE-101 24 Stockholm, Sweden

**Keywords:** Pheromone, Cattle, Oestrus, Oestrous synchrony

## Abstract

**Background:**

Declining fertility is a major concern for dairy farmers today. One explanation is shorter and weaker expression of oestrus in dairy cows making it difficult to determine optimal time for artificial insemination (AI). Chemical communication is of interest in the search for tools to detect oestrus or to synchronise or enhance oestrous periods. Pheromones, used in chemical communication within species, can influence reproduction in different ways. The aim here was to investigate whether oestrous cycle length, and duration and intensity of oestrous expression in dairy heifers could be manipulated through exposure to pheromones in oestrual substances from other females.

**Methods:**

Beginning on day 16 of two consecutive control oestrous cycles, ten heifers of the Swedish Red Breed (SRB) were exposed to water. During the two following cycles the heifers were exposed to urine and vaginal mucus, obtained from cows in oestrus. Cyclicity parameters were monitored through hormone measurements, oestrus detection and ultrasonographic examination.

**Results:**

We found no difference in cycle length or in duration of standing oestrus between control and treatment. We did, however, find a tendency of interaction between type of exposure (control or treatment) and cycle number within type of exposure for cycle length (p = 0.068), with the length differing less between the treatment cycles. We also found a tendency of effect of type of exposure on maximal concentration (p = 0.073) and sum of concentrations (p = 0.063) of LH during the LH surge, with values being higher for the control cycles. There were also significant differences in when the different signs of oestrus occurred and in the intensity of oestrous expression. The score for oedema and hyperaemia of external genitalia was significantly higher (p = 0.004) for the control cycles and there was also a significant interaction between type of exposure and time period for restlessness (p = 0.011), with maximum score occurring earlier for treatment cycles.

**Conclusions:**

No evidence of altered oestrous cycle length or duration of oestrus after exposure of females to oestrous substances from other females was found. Expression of oestrus, and maybe also LH secretion, however, seemed influenced by the exposure, with the effect of treatment being suppressive rather than enhancing.

## Background

Through breeding programs and improved management, the dairy cow milk yield has increased substantially since the 1950s. At the same time the dairy cow reproductive performance is deteriorating worldwide, which is partly attributable to the negative genetic correlation between milk yield and fertility [1]. One explanation for the poor reproductive results may be that the expression of standing oestrus is growing weaker and shorter [2], resulting in faltering oestrus detection [3,4] and, consequently, poorly timed artificial insemination (AI). This decline in fertility brings substantial economic losses to farmers [5].

A synchronised breeding period in the herd, which can be achieved through the use of exogenous hormones, makes the use of AI more efficient [6]. However, in many countries the consumers show reluctance to accept the use of hormones in healthy animals and there is a strong trend for limiting the use of exogenous reproductive hormones in Northern Europe. The use of non-hormonal substances for the same purpose may be more acceptable.

Pheromones are chemical substances, used for communication within species, which elicit a specific reaction in the receiver [7], either an immediate behavioural effect (releaser pheromones) or slower neuroendocrine changes (primer pheromones) [8,9]. Due to these effects, pheromones are good potential candidates for oestrous synchronisation and may also be used to detect oestrus or to reinforce oestrous behaviour in suboestrual animals.

Several studies show that pheromones affect reproduction in many domestic animals and also in humans. Pheromones can, among other things, induce mating behaviour, hasten puberty and shorten periods of anoestrus (see [10,11] for reviews). Inter-female pheromones can also cause modifications of the oestrous cycle [12]. One example of oestrous cycle modification is oestrous synchrony, which has been widely debated over the last few decades. There is evidence that volatiles from females can cause synchronisation of oestrous cycles, i.e. in rats [13] and in humans [14,15]. Izard and Vandenbergh [16] found indications that the same might apply to dairy heifers by exposing heifers to oestrous urine and vaginal discharge after treatment with prostaglandin F2α (PGF2α). In this last study, exposure to vaginal discharge caused a higher degree of oestrous synchrony. According to Kiddy & Mitchell [17] oestrous odour in fluids from the bovine reproductive tract increases slowly during the three days before oestrus and disappears within one day after oestrus.

Earlier studies indicate that bovine oestrual urine and vaginal mucus may contain pheromones, which can induce and/or stimulate male sexual behaviour [18-20] and influence other females [16,21]. However, only a handful studies on chemical communication between females have been published and proof that inter-female pheromones actually exist and can cause synchronisation of oestrous cycles or enhancement of oestrous behaviour in cattle is still lacking. Aron [11] proposed three mechanisms through which the oestrous cycle could be manipulated by exposure to pheromones; through stimulation of the neuroendocrine structures controlling the corpus luteum, by changing the follicular growth rate or by inducing an ovulatory LH release. The focus here was the follicular phase and the objective was to investigate, in a strictly controlled manner, the influence of oestrual substances on the oestrous cycle length, endocrinology, ovarian follicular development and oestrous behaviour in cattle. For this we used substances collected within 48 h from ovulation. This is in accordance with the findings of Kiddy & Mitchell [17] and includes, but is not limited to, substances collected during the period of standing oestrus.

## Methods

### Experimental animals

Ten cyclic heifers of the Swedish Red breed were housed individually in separate isolated rooms at the Swedish University of Agricultural Sciences (SLU), Uppsala, Sweden. The first two heifers took part in the study during the autumn of 2006, the next four during the autumn of 2007 and the final four during the spring of 2008. The rooms were adjacent, located pair wise, and each room had a separate entrance. The heifers could not see each other, but they shared the same ventilation system and could, to some extent, hear each other. Changing of clothes (coats, boots and head wear) and washing of hands were mandatory before entering each room. The heifers were kept in tie stalls, where they were fed straw ad libitum and limited amounts of concentrate. The average age of the animals was 15.9 ± 0.7 months (mean ± SD, range 14.9-16.9 months) at the start of the experiment, and the average body weight was 353 ± 41 kg (mean ± SD, range 293-396 kg). All animals were examined with transrectal ultrasound to confirm ovarian cyclicity, i.e. an active corpus luteum in the ovaries, before they entered the study. The first oestrus in the study was induced through one or more intramuscular injections of 0.5 mg of PGF2_α _(Cloprostenol sodium, Estrumate^®^, Intervet, Boxmeer, Netherlands).

### Donor animals

Vaginal mucus and urine were collected from cyclic heifers and cyclic, non-lactating cows of the Swedish Red and the Swedish Holstein breeds housed at SLU. These animals were not the same as the experimental animals mentioned above. The animals were housed in conventional tie stalls and fed hay ad libitum. In the donor animals, oestrus was induced through one or more intramuscular injections of 0.5 mg of PGF2_α_.

### Vaginal mucus and urine

Collection of substances from the donor animals started when at least one follicle larger than 12 mm was observed in the ovaries following the last injection of PGF2_α _(i.e. approximately 48 hours later). The collection continued until ovulation. During this period the animals' ovaries were monitored with transrectal ultrasound and visual oestrus detection was performed at least twice daily, at the beginning and the end of day (8-10 h apart). The ultrasonographic examinations of the ovaries were made using a portable ultrasound (Agroscan ALR 757, ECM, Angoulème, France) with a 7.5 MHz linear rectal probe. Ovulation time was determined as the midpoint between the last observation of the ovulatory follicle and the first observation of the ovulated ovary. Urination was induced, once during the 48 hours, by manual stimulation of the perineum and the urine was collected in a glass vial. It was then poured into plastic vials (polypropylene) and stored at -80°C. The vaginal mucus was collected using tampons hand manufactured by the authors, out of clean gauze and cotton string, for the purpose of this study, to avoid chemical substances as in commercially available tampons. The tampons were placed in the cranial vagina through a speculum and left there for 1-2 hours before they were removed and placed in plastic vials at -80°C. The substances collected earlier than 48 hours before ovulation or after ovulation were discarded.

In total, 231 urine samples, collected from 15 animals, and 231 mucus samples, collected from 20 animals, were used for exposure. The samples were, to equal proportions, collected before, during and after oestrus. The individual experimental heifers were exposed to substances from different animals and different stages during each treatment cycle.

### Experimental design

**Figure 1:**
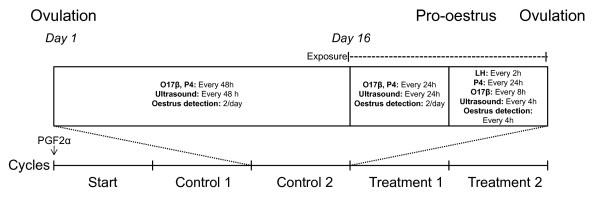
**Experimental design**. A schematic view of all five cycles, where the second control cycle is shown in detail. All four experimental cycles followed the same schedule concerning blood sampling for oestradiol (O17β), progesterone (P_4_) and luteinising hormone (LH), ultrasonographic examination of the ovaries and oestrus detection.

The experiment started with a start cycle, for which the purpose was to register the time point of the peak (LH peak) of the first LH surge (the period of elevated LH concentration) and ovulation for calculating the oestrous cycle length (expressed as LH peak interval or ovulatory interval). The time point of the LH peak was defined as the midpoint between the two blood samples with the highest LH concentrations during each surge. After the start cycle followed the four experimental cycles, starting with two control cycles and ending with two treatment cycles (Figure 1). Induction of oestrus through administration of PGF2_α _was only performed at the onset of the start cycle and all subsequent oestruses were spontaneous. The same procedure was repeated for all four experimental cycles, with the exception of exposure to water during the control cycles and to both urine and vaginal mucus during the treatment cycles. Hence, each animal was its own control.

From day 1 (day of ovulation) until day 15 of the experimental cycles, blood samples were taken every second day. The blood samples were collected into heparinised glass tubes (Venoject, Terumo Europe N. V., Leuven, Belgium) and centrifuged at 3000 × g for 10 minutes. The plasma was then removed and stored in plastic vials at -20°C until analysis. From day 16 until pro-oestrus (increasing restlessness, oedema and hyperaemia of external genitalia and cloudy vaginal discharge) the animals were bled once daily. Before the onset of pro-oestrus, the animals were fitted with either an indwelling silicone tube (ref. 602 285, SEDAT, Irigny, France) or a central venous catheter (ref. CVP70255, Surgivet, Smiths Medical Pm Inc., Waukesha, USA) in the jugular vein. During the procedure, the animals were sedated with an intravenous injection of 0.05 mg/kg of xylazine (Narcoxyl^®^, Intervet, Boxmeer, Netherlands) and local anesthesia was induced with 200 mg of lidocain (Xylocain^®^, AstraZeneca PLC, London, Great Britain). From the onset of pro-oestrus blood was collected every second hour until ovulation had occurred.

Oestrus detection was performed twice daily from the beginning of the oestrous cycle until the onset of pro-oestrus. The animals were also monitored with ultrasound examination of the ovaries, first every other day and then daily from day 16. From the onset of pro-oestrus until ovulation both ultrasonographic examination and visual oestrus detection were performed every four hours.

**Figure 2:**
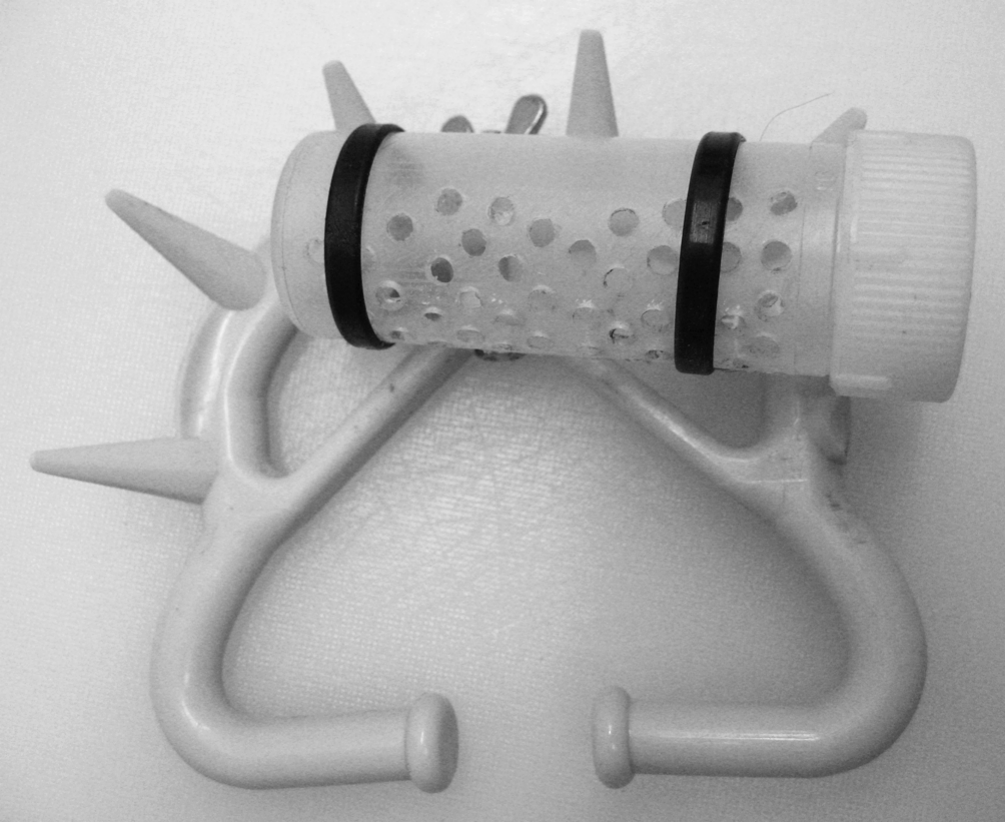
**Modified nose ring with the plastic cassette for holding the tampon attached**.

On day 16 of the oestrous cycle the animals were fitted with a non-invasive nose ring originally designed to prevent unwanted suckling (Cattle Weaner Müller, Albert Kerbl GmbH, Buchbach, Germany). The experimental substances were presented to the animals on similar cotton tampons as described above, placed in a plastic cassette attached to the nose ring (Figure 2). The frozen substances were thawed in a water bath (40°C for 45 minutes) and then kept in an isolated box at approximately 45°C while being distributed. For the vaginal discharge the collected tampons were used as they were and for the urine and water 10 mL was poured onto a fresh tampon. Before the tampons were put in the cassette, they were also inserted into the nasal cavity of the animal. The tampons were replaced with fresh ones at 12-hourly intervals.

All animal experiments described in this paper were approved by the Uppsala regional ethical committee (approval no. C160/6) and carried out in accordance with EC Directive 86/609/EEC.

### Selection and limitations of data

The protocol for the first group of two heifers did not include the intensive sampling of the start cycle, but it did for the other two groups. Also, for that group the equipment for ultrasonographic recordings was not available, so these animals were left out of the retrospective dynamics analysis. Furthermore, one heifer in the third group had to be replaced after the start cycle. The start cycle of the new animal occurred at the same time as the first control cycle of the other animals and was followed by only one control cycle, i.e. control cycle 1.

The expression of pro-oestrus varied between the animals, making it difficult to start the intensive blood sampling at a standardised time point. To be able to use data from as many cycles as possible, the inclusion of data had to be standardised in accordance with the cycles with the lowest number of blood samples and therefore many blood samples were excluded (see 'Statistical analysis' for further explanation).

### Oestrus detection and scoring scale

To evaluate duration of oestrus and strength of expression, the visual oestrus detection was performed in a standardised manner using a scoring scale (Table 1), modified from van Eerdenburg et al. [22]. The total duration of each oestrus detection event was 3 minutes. During this time five signs of oestrus were evaluated and scored: position (standing, getting up or lying), restlessness (strong, average or calm), lordosis (induced by eye contact, approach or touch), vaginal discharge (clear with high viscosity or cloudy with low viscosity) and appearance of external genitalia (red and/or swollen). The range of the scoring scale for each of the five signs was set according to the estimated relative importance of the sign. The sum of the scores for the five traits could range from 0-29 points. The onset of standing oestrus was recorded at the midpoint between the first oestrus detection when the animal displayed lordosis and the following oestrus detection. Conversely, the end of standing oestrus was recorded at the midpoint between the last oestrus detection where lordosis was displayed and the subsequent detection. This definition of oestrus has been used before when monitoring oestrous behaviour in tie stalls [23,24]. Lordosis in tied animals has been shown to be consistent with standing to be mounted by the bull [23], which is considered to be the only reliable sign of true oestrus.

**Table 1:** Scoring scale for oestrus detection

Oestrous sign	Scoring scale
*Position:*	

Standing	3

Getting up	1

Lying	0

*Restlessness:*	

Strong	3

Average	1

Calm	0

*Lordosis:*	

At eye contact	15

At approach	12

At touch	10

None	0

*Vaginal discharge:*	

Clear	5

Cloudy	2

Bloody or none	0

*External genitalia:*	

Red and swollen	3

Red or swollen	1

Normal	0

### Follicular dynamics in the experimental animals

The ultrasonographic examinations of the ovaries were made using the same portable ultrasound as above, now fitted with a digital recording device (PMP-100, Sigmatek, France). The size of the ovulatory follicle was recorded and it was traced back to the ultrasound examination on day 16 (the start of exposure) or, if it occurred later, to when it attained a size of 7 mm or larger, which should be close to deviation [25]. The mean growth rate per day, from either the ultrasound examination on day 16 or from when it became ≥ 7 mm to ovulation, was then calculated.

### Hormonal profiles in the experimental animals

Aliquots of the blood samples were stored at -20°C for the duration of the experiment and were then sent in one single batch for each hormone to the laboratories responsible for the analyses. Analysis of LH and progesterone were carried out at the University of Liège, Belgium, and oestradiol analysis was performed at the University of Agricultural Sciences, Sweden. All analyses were carried out by the staff of the laboratories.

Blood samples taken at two-hourly intervals between 34 and 14 h before ovulation were analysed for LH to identify the preovulatory LH peak. Plasma concentrations of LH were determined using a double antibody radioimmunoassay (RIA) procedure, as described previously [26]. The minimum detection limit of the LH-RIA technique was 0.45 ng/mL. The within and between assay coefficients of variation (CV) were 5.1% (6.9 ± 0.4 ng/mL) and 11.8% (6.6 ± 0.8 ng/mL), respectively.

Progesterone (P_4_) concentrations were determined in blood samples taken once every second day during day 1-15 of the oestrous cycle and thereafter once daily until ovulation. The method used was a direct RIA method without extraction (Coat-A-Count, Siemens Healthcare Diagnostics Inc), as previously described in detail [27]. The minimum detection limit of the P_4_-RIA technique used was 0.48 nmol/L and within and between assay CVs of 13% (8.3 ± 1.3 nmol/L) and 19% (8.6 ± 1.6 nmol/L), respectively.

Oestradiol-17β was measured in blood samples taken every second day during day 1-15 of the oestrous cycle, thereafter once daily and, finally, from the onset of pro-oestrus until ovulation three times daily. Plasma concentrations of oestradiol-17β were determined by using a ^125^I-RIA (Double Antibody Estradiol, KE2D1, Siemens Healthcare Diagnostics Inc), previously validated for bovine plasma [28]. The samples were extracted with ether before analysis. The within and between assay CVs were 22.9 and 13.4% for the low control (5.5 pmol/L), 4.5 and 16.6% for the medium control (49.4 pmol/L) and 7.8 and 14.5% for the high control (148.5 pmol/L), respectively.

### Statistical analysis

Data were handled and analysed using SAS software (ver. 9, SAS Inst. Inc., Cary, USA).

For reproductive parameters and reproductive hormones, data were analysed by analysis of variance (ANOVA) using mixed models under a general linear model (GLM), the effect of the animal being considered as random. The statistical models included the fixed effects of type of exposure (2), cycle number within type of exposure (2) and the interactions between type of exposure and cycle number within type of exposure, between animal and type of exposure and between animal and cycle number within type of exposure, respectively. When the effect of the cycle number within type of exposure was found non-significant, this effect and corresponding interactions were removed from the model. For multiple comparisons following ANOVA, individual differences between means were assessed by Bonferroni tests. When needed, differences between estimates corresponding to each level of the significant factors and interactions with cycle number within type of exposure were also tested by using the contrast option.

For the sum of concentrations during the different blood sampling schemes (every 48 h during days 1-15, 24 h from day 16 to pro-oestrus and 8 h from pro-oestrus to ovulation for oestradiol and every 48 h during days 1-15 and 24 h from day 16 to ovulation for progesterone) the sums of the values from all measurements during each sampling period were calculated. For the 8 h sampling of oestradiol the last 3 samples before ovulation (i. e. approximately from the onset of standing oestrus to ovulation) were used. For the sum of concentrations during the LH surge the sum of the values from seven measurements, taken 26-13 h before ovulation, were calculated. The cycles, for which the number of blood samples available during the LH surge was lower than seven, were left out of the analysis.

When comparing the effect of the type of exposure on the expression of oestrus, the scoring data were divided into three different time periods; 0-14, 14-26 and 26-38 hours before ovulation. This grouping was based on the mean onset and end of standing oestrus for all cycles (n = 37) with oestrus starting and ending approximately 26 h and 14 h, respectively, before ovulation. The variation in the scoring data was analysed using analysis of variance (PROC MIXED). The statistical model included the fixed effects of type of exposure (2), cycle number (4) nested within type of exposure, time period (3) and the interaction between type of exposure and time period. The random effect of animal was also included in the statistical model. Least-squares means were calculated, and compared using student's *t*-test.

### Reproductive health of experimental heifers

At the end of the experiment six of the animals were inseminated and four underwent embryo transfer. They were then sent to a research farm with commercial dairy production. Fertility data for the animals were later collected from the farm records.

## Results

### Reproductive parameters

There were no significant differences between control and treatment groups with regard to the reproductive parameters that were investigated. A large variation was seen for several parameters. The means and standard deviations for control and treatment, respectively, are given in Table 2.

**Table 2:** Comparison of reproductive parameters (mean ± SD) between control and treatment groups

Parameter	Control		Treatment	
	
	N	Mean ± SD	N	Mean ± SD
Ovulatory interval (days)	17	20.1 ± 1.5	20	20.2 ± 1.2

LH peak interval (days)	15	20.2 ± 1.5	18	20.4 ± 1.1

Growth rate of dominant follicle (mm/day)	15	0.9 ± 0.3	16	0.9 ± 0.5

Max. oestradiol conc. to onset of standing oestrus^a ^(h)	18	3.2 ± 9.5	19	5.6 ± 8.7

Onset of standing oestrus^a ^to LH peak (h)	18	2.0 ± 4.9	19	0.42 ± 6.6

Onset of standing oestrus^a ^to ovulation (h)	18	26.9 ± 4.8	19	25.3 ± 7.0

Duration of standing oestrus^a ^(h)	19	12.2 ± 5.3	20	10.9 ± 5.4

End of standing oestrus^a ^to ovulation (h)	18	14.1 ± 4.4	19	13.8 ± 5.0

LH peak to ovulation (h)	19	24.9 ± 1.9	19	24.9 ± 2.1

Size of ovulatory follicle (mm)	19	13.1 ± 1.4	20	12.6 ± 1.2

^a^Standing oestrus defined as the time period when the animal displays lordosis. Difference between treatments: For all parameters p > 0.100. LH = luteinising hormone

**Figure 3:**
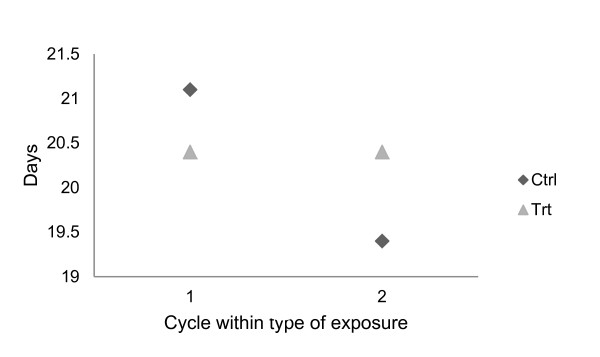
**Oestrous cycle length**. Least square means of luteinising hormone (LH) surge interval for each cycle within type of exposure. Ctrl = control cycle; Trt = treatment cycle.

We did find a significant effect of cycle number within type of exposure for cycle length, calculated both as ovulatory interval (p = 0.038) and as LH peak interval (p = 0.027), and a tendency for interaction between cycle number within type of exposure and type of exposure (p = 0.068 and 0.073 respectively) for both parameters. The length of the two treatment cycles was very similar (p = 0.95), while it differed significantly between the two control cycles (p = 0.042) as illustrated by LH surge interval in Figure 3.

### Reproductive hormones

There was no significant influence of the two types of exposure either on progesterone or on oestradiol concentration. There was, however, a tendency for a treatment effect on both the sum of concentrations of LH during the surge (p = 0.063) and on the peak concentration of the LH surge (p = 0.073), with higher values for the control cycles. Means and standard deviations for all parameters are given for control and treatment, respectively, in Table 3.

**Table 3:** Comparison of hormonal parameters (mean ± SD) between control and treatment groups

Parameter		Control		Treatment	
		
		N	Mean ± SD	N	Mean ± SD
Progesterone (nmol/L):	Sum of 48 h-samplings^a^	17	73.8 ± 17.6	20	80.2 ± 38.5

	Sum of 24 h-samplings^a^	19	42.2 ± 31.6	20	44.6 ± 29.9

Oestradiol (pmol/L):	Sum of 48 h-samplings^a^	17	40.2 ± 14.1	20	34.4 ± 14.9

	Sum of 24 h-samplings^a^	19	78.6 ± 26.0	20	71.8 ± 21.5

	Sum of 8 h-samplings^a^	19	27.8 ± 11.1	20	25.6 ± 14.5

	Max. concentration	19	34.8 ± 8.6	20	35.2 ± 15.0

LH (ng/mL):	Sum of measurements during surge^a, b^	19	68.6 ± 21.3	19	60.9 ± 18.2

	Peak concentration^c^	19	26.8 ± 12.5	19	22.1 ± 7.3

^a^The sum of concentrations during the different blood sampling schemes. Difference between treatments: ^b^p = 0.063; ^c^p = 0.073. For all other parameters p > 0.100. LH = luteinising hormone

**Figure 4:**
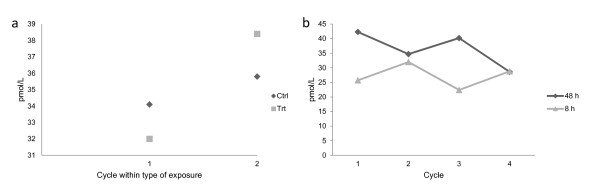
**Oestradiol concentration**. A, Least squares means of maximum oestradiol concentration for each cycle within type of exposure. Ctrl = control cycle; Trt = treatment cycle. B, Least squares means of the sums of concentrations of oestradiol during the 48 h and 8 h sampling, respectively, for the four experimental cycles. Cycles 1-2 are control cycles and cycles 3-4 are treatment cycles.

Significant effects of the cycle number within type of exposure were observed on maximum oestradiol concentration (p = 0.049), the sum of oestradiol concentrations during the 48 h sampling (p = 0.009) and during the 8 h sampling (p = 0.020). The maximum concentration of oestradiol was quite similar between the two control cycles, while it differed more between the two treatment cycles (Figure 4a). The pattern of all four cycles for the sum of oestradiol concentrations during days 1-16 (48 h-sampling) seemed to be an inversion of the pattern seen during the last 24 h before ovulation (8 h-sampling). This effect is illustrated in Figure 4b.

### Scoring scale for oestrus detection

**Figure 5:**
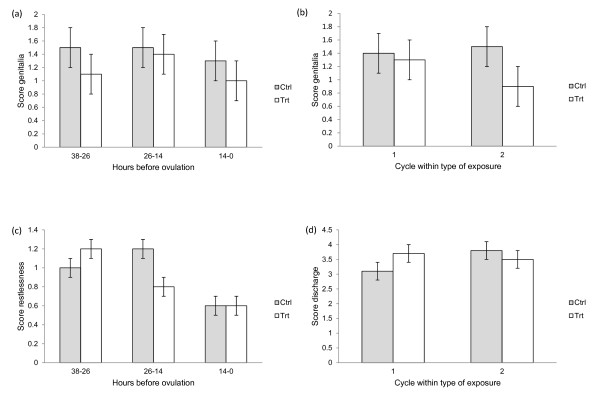
**Oestrus scoring scale data**. Least squares means and standard errors of the score, for control (Ctrl) and treatment (Trt) respectively, for a, genitalia during three time periods preceding ovulation; b, for genitalia for first and second cycle within type of exposure; c, for restlessness during three time periods preceding ovulation; d, for discharge for first and second cycle within type of exposure.

The number of observations within each time period (0-14, 14-26 and 26-38 h before ovulation) was quite similar. The effect of time period on the score was highly significant (p < 0.001) for all variables except for appearance of genitalia (p = 0.025). The score for genitalia was significantly higher (p = 0.004) during the control cycles than during the treatment cycles (Figure 5a). There was also a significant effect of cycle number within type of exposure (p = 0.007), illustrated in Figure 5b. The score increased in the second control cycle, while it decreased in the second treatment cycle. There was a significant interaction between type of exposure and time period for restlessness (p = 0.011; illustrated in Figure 5c) and a tendency for such an interaction for position (p = 0.076). During the treatment cycles the maximum score for both restlessness and position occurred before the onset of standing oestrus (26-38 h before ovulation), while it in the control cycles occurred during standing oestrus (14-26 h before ovulation). For the variable discharge there was an effect of cycle number within type of exposure (p = 0.026), following the same pattern as for genitalia with increased scores for the second control cycle and decreased for the second treatment cycle (Figure 5d) For the variables lordosis and total score there were no significant effects other than that of time period.

### Reproductive health of experimental heifers

The pregnancy rate after first insemination or embryo transfer was 90% and eight of the nine pregnant animals gave birth to live calves.

## Discussion

There is anecdotal evidence from farmers that cyclic animals grouped together tend to synchronise their oestrous cycles and the study of Izard and Vandenbergh [16] supports this. However, no study has ever provided indisputable proof of inter-female primer pheromones causing synchronisation of oestrous cycles in cattle. This could be because there is no such pheromone or because the complexity of large mammals makes it difficult to design and carry out the necessary experiments. In the present study, unique in that it was performed on animals in isolation, we found no effect of oestrous urine and vaginal mucus on the cycle length, neither when measured as the interval between LH surges nor as the interval between ovulations.

There are several possible explanations why there was no significant effect of the type of exposure on oestrous cycle length, which was the underlying hypothesis of this study.

Firstly, there is of course the possibility that no oestrous synchrony pheromone exists. We did, however, see differences in expression of oestrus and also a tendency for an effect on LH concentration between the two types of exposure, which may be caused by the exposure to oestrous substances, so the hypothesis of a bovine inter-female pheromone cannot be rejected. However, the two treatment cycles investigated in the present study may not be enough time for the effect to fully manifest. In the present study we focused on the follicular phase. Investigating the transition between luteal and follicular phases, in particular the release of prostaglandin, might have provided additional information and is of great interest for future studies.

Secondly, there may be a risk that bioactivity of the samples was lost during the handling and storage of the samples, either through evaporation or through degradation. Studies on bovine vaginal mucus [18] and female elephant urine [29], however, indicate that this risk is slight under present conditions.

Thirdly, our assumption was that the cycle length within an unexposed animal is constant. It is possible that the inter-cycle variability is greater than presumed, which would prevent the detection of any influence of treatment on the cycle length. We did, however, find a significant effect of cycle on peak interval, which was due to the difference in length of the control cycles. These different patterns are difficult to explain, but they show that an effect of the treatment cannot be rejected.

Fourthly, the theoretical coupled-oscillator model for oestrous synchrony that has been described previously in humans and in rodents [15,30,31], includes two different pheromones with adverse actions, one that shortens the cycle length and a second that has a lengthening effect. In humans, the first pheromone is thought to be released during the follicular phase (2-4 days before the preovulatory LH surge) and the second during the ovulatory phase (the day of the LH surge to two days after) [15]. In the present study samples were collected during the 48 h preceding ovulation, i.e. both before and after the preovulatory LH surge. If the coupled-oscillator theory is true for the cow, this may have caused a counteraction mechanism, masking any effect on the cycle length. It is imperative to state, however, that this theoretical model [32] and oestrous synchrony as such has been called into question [33].

Finally, there may also be a synergistic effect between pheromones and other biostimuli, i.e. that it's not only the substance that is needed for the effect to manifest, but also the oestrous behaviour of the other animal. Since the animals in the present study were kept isolated, they were not exposed to such visual or tactile stimuli from other animals. Another possibility is that the effect is mediated by a signature mixture, rather than a pheromone. A signature mixture is an individual-specific mix of chemical signals that need to be learnt by the receiver [34]. Nishimura et al. [21] demonstrated that heifers, smeared with their own oestrual discharge during dioestrus, were nearly always mounted by their herd mates, while dioestrual heifers, that were smeared with the oestrual discharge of another animal, were not. These results support the theory of a signature mixture, rather than a pheromone, acting between females, which could explain the lack of an effect on the cycle length in the present study, where the animals were exposed only to the substances. It may also be that different animals release different amounts of pheromone, so that a threshold concentration is not surpassed, or that the receptivity of the receivers may vary. The limited numbers of experimental animals and, to some extent, donor animals would make the present study vulnerable to such variability.

We did see a tendency for an effect of type of exposure on the preovulatory LH surge, as suggested by Aron [11]. However, this tendency concerned the peak LH concentration and sum of LH concentrations during the surge, with higher values for the control cycles, and not the timing of the surge. In a previous study we investigated the effect of oestrous urine and vaginal discharge on the LH pulsatility pattern preceding the preovulatory surge and found that the pattern differed significantly between the two types of exposure, with increased nadir concentrations and decreased amplitude of peaks during treatment cycles [35]. It is possible that there really is an inhibiting effect of treatment on the two LH parameters, but that the number of animals in this study is too limited for this difference to manifest significantly. Moreover, the large variation in several of the hormonal and reproductive variables might, to some extent, also explain the lack of differences between the two types of exposure.

The effect of cycle within type of exposure for maximum oestradiol concentration is interesting, indicating that control cycles are more homogenous and that the variability of this parameter is greater during the treatment cycle.

Even though neither the duration nor the strength of expression of oestrus (measured as the total score) differed between the two types of exposure, we found a significant interaction between time period and type of exposure for the variable restlessness. During the control cycles increased restlessness occurred mainly during the period of standing oestrus (14-26 h before ovulation), while it occurred earlier (26-38 h before ovulation) during the treatment cycles. There were also indications that the same may apply to the variable position. The score for genitalia showed the opposite pattern with similarly high scores during the first two periods for the control cycles and the maximum score for the treatment cycles occurring during the period of standing oestrus. These effects might be caused by endocrinological changes due to the exposure, even though we could not detect such differences in our data.

The score for genitalia and discharge increased between control cycles 1 and 2, while it decreased between the treatment cycles. This cycle effect might be caused by the long time in isolation. Such an effect could have been avoided by randomising the order in which the two types of exposure occurred. However, due to the unexplored nature of bovine primer pheromones the present schedule was chosen to avoid a possible spillover effect from periods of treatment to periods of control and also to avoid contamination when handling the substances simultaneously. It may also be that the treatment has an additional or delayed effect, so that the effect can't be seen until the last cycle of the treatment.

## Conclusions

Exposing heifers to oestrous urine and vaginal mucus caused no effect on the length of the oestrous cycle. However, the length of the two treatment cycles was very similar, while it differed significantly between the two control cycles. We did also see an effect on the expression of oestrus, which may have been caused by an inter-female pheromone. Furthermore, a tendency for an effect on the maximal LH concentration and the sum of concentrations of LH during the preovulatory LH surge was seen, which further support our previous findings regarding the LH pulsatility pattern preceding the preovulatory LH surge [35]. We believe that further studies, focusing on the LH secretion, on a larger number of animals would be of great interest for future research on bovine inter-female pheromones.

## Abbreviations

AI: Artificial insemination; ANOVA: Analysis of variance; CV: Coefficient of variation; GLM: General linear model; LH: Luteinising hormone; PGF2_α_: Prostaglandin F2_α_; RIA: Radioimmunoassay; SLU: Swedish University of Agricultural Sciences.

## Competing interests

The authors declare that they have no competing interests.

## Authors' contributions

KN participated in the design of the study, carried out all experimental procedures and drafted the manuscript. RB participated in the design of the study and helped to draft the manuscript. HG participated in the design of the study and helped to draft the manuscript. PH performed the statistical analyses, except for the oestrus scoring scale data, and commented on the manuscript. LS participated in the design of the experiment and helped to draft the manuscript. All authors read and approved the final version of the manuscript.
